# Activating Triplet Excitons in COFs via Layer‐Bridging Co Single Atoms for Photocatalytic H_2_O_2_ Production and Tandem C─N Coupling

**DOI:** 10.1002/anie.1580812

**Published:** 2026-08-19

**Authors:** Guoen Tang, Jianjun Zhang, Guijie Liang, Jinfeng Zhang, Chuanbiao Bie, Jiaguo Yu, Hermenegildo García

**Affiliations:** ^1^ Laboratory of Solar Fuel Faculty of Materials Science and Chemistry China University of Geosciences Wuhan P. R. China; ^2^ Hubei Key Laboratory of Low Dimensional Optoelectronic Materials and Devices Hubei University of Arts and Science Xiangyang Hubei P. R. China; ^3^ College of Physics and Electronic Engineering Huaibei Normal University Huaibei P. R. China; ^4^ Instituto Universitario de Tecnología Química, CSIC‐UPV Universitat Politècnica de València Valencia Spain

**Keywords:** covalent organic frameworks, energy‐level splitting, single‐atom engineering, spin–orbit coupling, triplet excitons

## Abstract

Covalent organic frameworks (COFs) are promising photocatalysts for solar energy conversion in energy and environmental applications. However, their performance is intrinsically limited by weak spin–orbit coupling (SOC) arising from van der Waals interlayer interactions, thereby suppressing intersystem crossing and triplet‐exciton utilization. In this study, we demonstrate that layer‐bridging Co single atoms activate triplet excitons in COFs by synergistically enhancing SOC and inducing energy‐level splitting via Co─N coordination. This layer‐bridging Co coordination markedly promotes singlet‐to‐triplet conversion, enabling efficient O_2_ activation and substantially boosting the generation of both ^1^O_2_ and O_2_^•−^. Consequently, the Co‐bridged COF achieves highly efficient photocatalytic H_2_O_2_ production and tandem C─N coupling. This work establishes layer‐bridging single‐atom coordination as a promising strategy for regulating triplet‐state reactivity in COFs, offering new opportunities for reactive oxygen species (ROS)‐mediated photocatalysis and tandem organic transformations.

## Graphical abstract

This work demonstrates a layer‐bridging coordination strategy of Co single atoms in COFs that promotes the formation and utilization of triplet excited states. The introduction of Co single atoms induces pronounced energy‐level splitting and enhanced spin–orbit coupling, facilitating the generation of ^1^O_2_ and O_2_^•–^ for efficient photocatalytic H_2_O_2_ production and tandem C─N coupling.

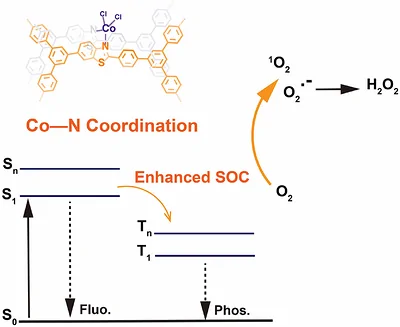

## 1 Introduction

Harnessing solar energy to drive chemical transformations is central to achieving carbon neutrality, placing stringent demands on the efficiency and selectivity of photocatalysts [1, 2, 3]. Covalent organic frameworks (COFs) have emerged as promising platforms for photocatalytic reactive oxygen species (ROS) generation owing to their periodic architectures [4, 5], modular structural tunability [6, 7], and strong visible‐light absorption [8]. Nevertheless, the extended π‐conjugation in COFs leads to tightly bound excitons with high binding energies [9]. Although high exciton binding energies can, in principle, favor energy‐transfer pathways (e.g., ^1^O_2_ generation) in certain molecular systems, in crystalline COFs they lead to strong exciton localization and limited exciton mobility, along with a large singlet–triplet energy gap (Δ*E*_ST_) and intrinsically weak spin–orbit coupling (SOC). These factors collectively suppress efficient intersystem crossing (ISC), resulting in a low population of triplet excitons. As a consequence, triplet‐mediated ROS generation in COFs remains intrinsically inefficient.

Efficient photocatalytic ROS generation relies critically on ISC from singlet excited states to long‐lived triplet excitons, which can sensitize molecular oxygen to produce singlet oxygen (^1^O_2_) or participate in electron transfer to produce superoxide radicals (O_2_^•−^). Because ISC is intrinsically limited by spin‐forbidden transitions, enhancing SOC represents a central strategy to activate triplet‐mediated pathways [10]. To this end, diverse approaches, including molecular engineering [11, 12], donor–acceptor architectures [13, 14], heteroatom incorporation [15, 16], and energy‐level splitting [17, 18], have been explored to promote SOC. However, none of these strategies simultaneously achieves effectiveness, cost efficiency, and scalability in crystalline COF systems. For instance, low‐cost sulfur heteroatom incorporation, while effective in small‐molecule systems [19, 20], typically induces only marginal SOC enhancement in extended COF lattices. By contrast, heavy‐metal‐based strategies employing third‐row elements such as Pt or Ir can significantly strengthen SOC but are hindered by high cost and poor scalability [21]. In this context, earth‐abundant transition metals emerge as an attractive alternative for regulating excited‐state dynamics. Among them, cobalt has recently demonstrated promising capability in modulating photophysical processes in molecular complexes [22, 23], yet its potential to regulate spin‐dependent excitonic processes in crystalline and extended COF frameworks remains largely unexplored.

Here, we demonstrate that layer‐bridging coordination of earth‐abundant Co single atoms provides an effective route to activate triplet excitons in COFs. By systematically comparing intralayer sulfur incorporation and layer‐bridging Co single‐atom coordination, we reveal that sulfur incorporation primarily promotes O_2_^•−^ formation without substantially enhancing SOC or triplet‐state generation. In sharp contrast, Co single atoms coordinated between adjacent COF layers synergistically enhance SOC and induce pronounced energy‐level splitting via Co─N coordination. Meanwhile, Co coordination moderately reduces the exciton binding energy, which facilitates the generation of free charge carriers. This dual modulation enables efficient singlet‐to‐triplet conversion and electron‐hole separation, leading to markedly enhanced generation of both ^1^O_2_ and O_2_^•−^. As a result, the Co‐bridged COF exhibits highly efficient photocatalytic H_2_O_2_ production and tandem C─N coupling. These findings establish layer‐bridging single‐atom coordination as an effective strategy to activate triplet excitons in COFs, offering new opportunities for ROS‐mediated photocatalysis and solar‐to‐chemical transformations.

## 2 Results and Discussion

### 2.1 Characteristics of the COFs

The parent imine‐linked COF (DTB), sulfur‐incorporated COF (SDTB), and Co‐single‐atom‐coordinated COF (SDTBCo) were synthesized sequentially to enable a controlled comparison between intralayer backbone heteroatom incorporation and layer‐bridging single‐atom coordination. DTB was obtained via a Schiff‐base condensation between 1,3,5‐tris(4‐aminophenyl)benzene (TB‐NH_2_) and 1,3,5‐tris(4‐formylphenyl)benzene (TB‐CHO). Sulfur incorporation was achieved through a post‐synthetic oxidative cyclization, converting imine linkages into thiazole units and yielding SDTB [24]. Subsequent layer‐bridging coordination of Co single atoms afforded SDTBCo through Co─N interactions. The corresponding structure models are illustrated in Figures 1a–c. Powder x‐ray diffraction (XRD) patterns of DTB, SDTB, and SDTBCo exhibit well‐defined diffraction peaks at 4.2°, 3.9°, and 4.1°, respectively (Figures 1d–f), indicative of high crystallinity. Pawley refinements show excellent agreement between experimental and simulated patterns, with all weighted‐profile (*R*_wp_) and profile (*R*_p_) *R*‐factor values below 10% [25], and all three structures are well described by concave AA‐stacked layered models in the monoclinic *P*_m_ space group. Importantly, the close similarity of the refined structures (Figure S1) confirms that neither intralayer sulfur incorporation nor layer‐bridging Co coordination disrupts the long‐range order of the COF frameworks.

**FIGURE 1:**
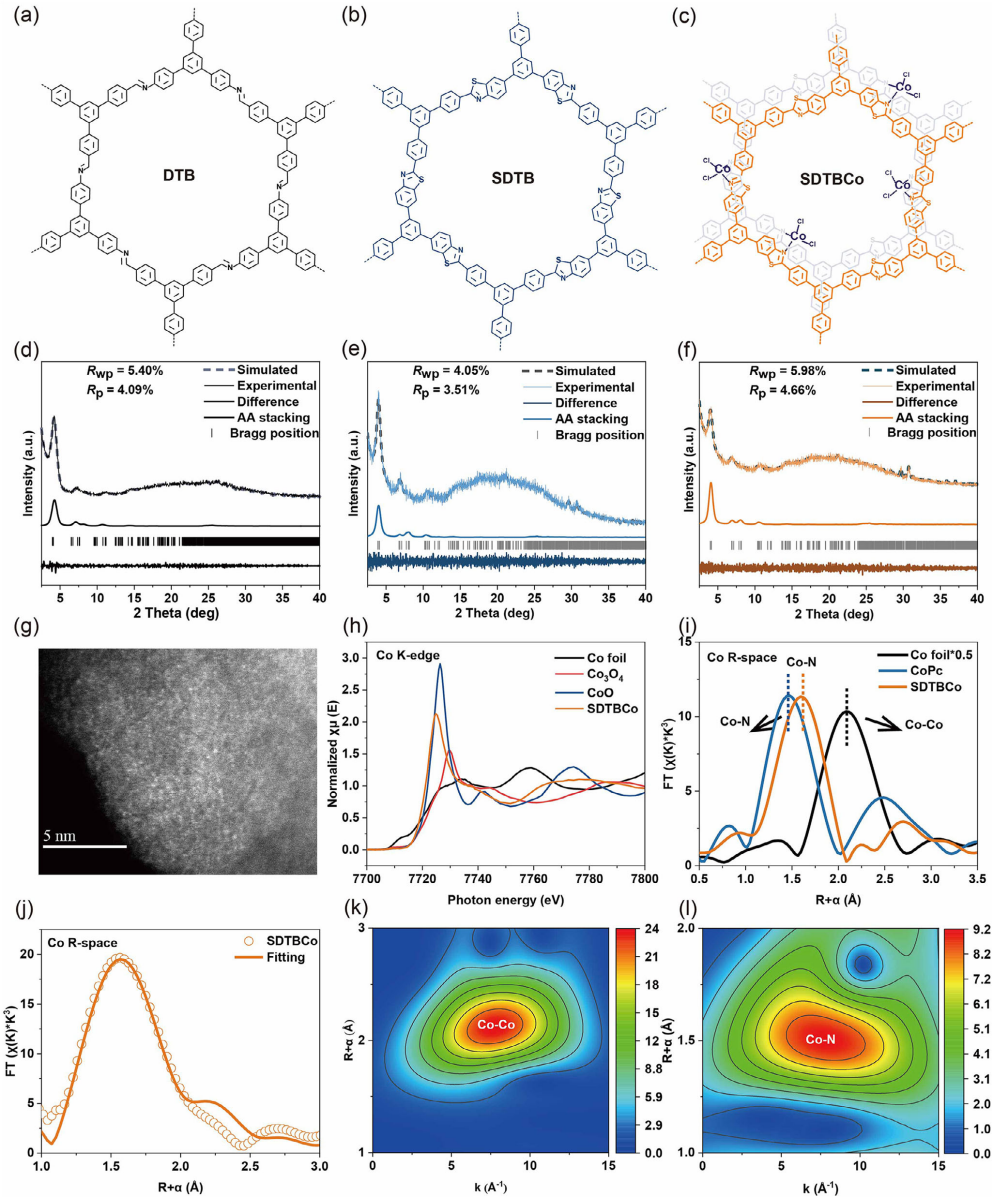
Structural characterization of COFs. Schematic representations of the crystal structures of (a) DTB, (b) SDTB, and (c) SDTBCo. Experimental and simulated XRD patterns of (d) DTB, (e) SDTB, and (f) SDTBCo. (g) Aberration‐corrected transmission electron microscopy (AC‐TEM) image of SDTBCo. (h) Normalized Co K‐edge x‐ray absorption near‐edge structure (XANES) spectra and (i) corresponding k^3^‐weighted Fourier‐transformed EXAFS spectra of Co foil and SDTBCo. The signal intensity of Co foil was multiplied by a factor of 0.5 for comparison purposes. (j) R‐space EXAFS fitting curves of SDTBCo. Wavelet transform‐EXAFS spectra of (k) Co foil and (l) SDTBCo.

Brunauer–Emmett–Teller (BET) analysis shows that SDTB and SDTBCo exhibit specific surface areas and pore‐size distributions comparable to those of DTB (Figure S2, Table S1), suggesting that all three COFs possess similar microscopic pore architectures. Consistently, field‐emission scanning electron microscopy (FESEM) reveals no discernible differences in external morphology among DTB, SDTB, and SDTBCo (Figure S3). Fourier transform infrared (FT‐IR) spectra further confirm successful framework construction (Figure S4). The characteristic N─H stretching vibrations of amino groups (3344–3435 cm^−1^) and C─H stretching vibrations of aldehyde groups (2825–2843 cm^−1^) [26] are absent in all three COFs, while a distinct stretching vibration at 1594 cm^−1^ corresponding to the ─N═C─ linkage [27] is clearly observed, indicating complete Schiff‐base condensation. Collectively, these results confirm that the observed differences in photophysical and photocatalytic behavior arise predominantly from electronic and spin‐related modulation rather than structural perturbation. X‐ray photoelectron spectroscopy (XPS) provides insights into the chemical environments of heteroatoms (Figure S5). In DTB, the N 1s spectrum can be deconvoluted into two components at 398.5 (N═C) and 399.7 eV (N─H) [28]. In SDTB, an additional N 1s component at 400.4 eV, ascribed to the N═C─S moiety [29, 30], emerges concomitantly with the characteristic S 2p peaks at 163.9 and 168.2 eV, confirming successful thiazole formation. In SDTBCo, these features are retained, while additional Co 2p signals (781.6, 785.6, 797.7, and 802.1 eV) characteristic of Co(II) species are observed, indicating successful incorporation of Co atoms without altering the backbone chemistry [31]. High‐resolution transmission electron microscopy (HR‐TEM) analyses reveal that DTB (Figure S6a,d), SDTB (Figure S6b,e), and SDTBCo (Figure S6c,f) exhibit comparable microcrystalline structures with no discernible morphological changes upon Co incorporation [32]. Moreover, no Co nanoparticles or clusters are detected in either HR‐TEM or AC‐TEM images (Figures 1g and S6c,f). Elemental mapping further confirms the homogeneous spatial distribution of Co throughout the SDTBCo framework (Figure S6g), providing direct evidence for the successful incorporation of Co single atoms.

The coordination environment of Co atoms in SDTBCo was elucidated by x‐ray absorption fine structure (XAFS) spectroscopy. In the Co K‐edge XANES spectrum, the absorption edge position of SDTBCo is located at higher energy than that of Co foil but lower than that of Co_3_O_4_, and closely resembles that of CoO (Figure 1h), indicating that the Co species predominantly exist in a valence state close to +2 [33]. The k^3^‐weighted Fourier‐transformed EXAFS spectra in R space are presented in Figure 1i. For Co foil, a prominent peak located at approximately 2.08 Å is observed, which can be assigned to Co─Co scattering. By contrast, this characteristic Co─Co peak is completely absent in SDTBCo, while a distinct peak centered at around 1.59 Å emerges, corresponding to the first coordination shell of Co─N bonds [34]. These results indicate that Co atoms in SDTBCo are atomically dispersed within the COF framework rather than aggregated into metallic clusters. Quantitative EXAFS fitting was performed independently for Co foil (Figure S7a,b and Table S2) and SDTBCo (Figures 1j and S7c,d; Table S2), revealing distinctly different local coordination environments. The absence of Co─Co coordination and the dominance of Co─N scattering paths in SDTBCo further substantiate the single‐atom dispersion of Co species. These differences are additionally corroborated by wavelet transform EXAFS analysis, in which the intensity maxima corresponding to Co─Co and Co─N contributions are clearly separated in K–R space for Co foil (Figure 1k) and SDTBCo (Figure 1l), respectively.

### 2.2 Enhanced Triplet‐State Dynamics and Oxygen Activation

The electronic structures of DTB, SDTB, and SDTBCo were systematically elucidated through combined spectroscopic analyses and theoretical calculations. Ultraviolet–visible diffuse reflectance spectra (UV–vis DRS, Figure S8a) show that, relative to DTB, SDTB exhibits an additional absorption feature (A1), which can be ascribed to sulfur‐induced intermediate energy levels [20]. Notably, SDTBCo not only retains this feature but also displays an additional absorption band (A2), characteristic of metal–ligand charge transfer (MLCT) [35], indicating strong electronic coupling between Co single atoms and the COF framework. Bandgap energies (*E*_g_) [36, 37, 38] extracted from Tauc plots (Figure S8b) were determined to be 2.50, 2.32, and 2.08 eV for DTB, SDTB, and SDTBCo, respectively, revealing a gradual narrowing of the bandgap upon sulfur incorporation and Co coordination. Mott–Schottky analyses (Figure S8c–e) further indicate that the estimated lowest unoccupied molecular orbital (LUMO) levels shift from −0.50 V (DTB) to −0.70 V (SDTB) and −0.82 V (SDTBCo) versus normal hydrogen electrode (NHE) [39, 40], based on the measured flat‐band potential (*E*_fb_) [41, 42]. Combined with the optical bandgaps, the corresponding highest occupied molecular orbital (HOMO) levels are calculated to be 2.00, 1.62, and 1.26 V, respectively.

Photoluminescence (PL) and time‐resolved photoluminescence (TRPL) spectroscopy were employed to elucidate the photogenerated charge‐carrier dynamics in the COFs. DTB (Figure 2a) and SDTB (Figure 2b) exhibit a single PL emission peak, which originates from the radiative recombination of photoexcited singlet‐state excitons to the ground state. In contrast, SDTBCo (Figure 2c) displays three distinct PL emission peaks. The PL1 peak originates from intrinsic band‐to‐band radiative recombination, as its emission energy is close to the optical bandgap. Notably, two additional anti‐Stokes‐shifted emission peaks (PL2 and PL3) [43] are observed, which can be attributed to MLCT‐assisted excitation processes [44]. The emergence of these anti‐Stokes emissions indicates the involvement of anti‐Kasha [45, 46] higher‐energy singlet excited states (*S*_n_) in SDTBCo (Figure S9). The population of such anti‐Kasha excited states is expected to increase the probability of ISC, thereby promoting triplet exciton generation. To verify this hypothesis, phosphorescence (PH) measurements were performed. All three COFs exhibit detectable PH (Figure S10), confirming the participation of triplet excitons. Importantly, PH lifetime measurements reveal that SDTBCo has the longest triplet‐state lifetime (2.70 ms), substantially exceeding those of SDTB (1.90 ms) and DTB (1.32 ms) (Figures 2d–f, Table S3). This result indicates more efficient singlet‐to‐triplet ISC and prolonged triplet exciton survival in SDTBCo, which is favorable for energy transfer to molecular oxygen.

**FIGURE 2:**
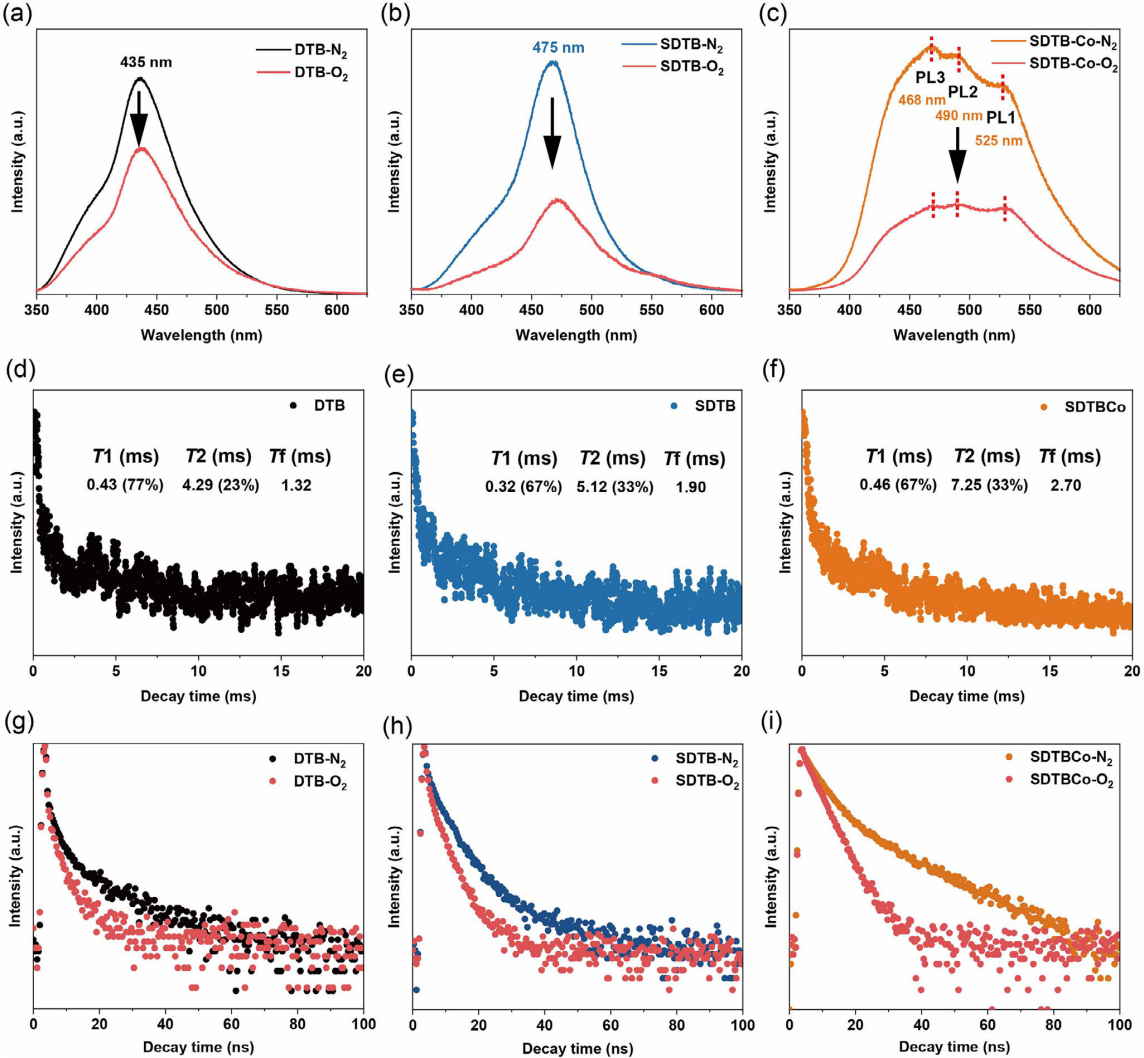
Photophysical evidence for enhanced triplet exciton dynamics. Steady‐state PL spectra of (a) DTB, (b) SDTB, and (c) SDTBCo measured under N_2_ and O_2_ atmospheres. Phosphorescence lifetime spectra of (d) DTB, (e) SDTB, and (f) SDTBCo under N_2_ atmospheres. TRPL spectra of (g) DTB, (h) SDTB, and (i) SDTBCo under N_2_ and O_2_ atmospheres.

Density functional theory calculations of O_2_ adsorption energies and charge‐transfer characteristics were performed (Figure S11). Compared with DTB, SDTB provides an increased number of sulfur‐containing sites capable of adsorbing O_2_ and donating electrons to it, as evidenced by the negative charge‐transfer values of O_2_, thereby facilitating its activation [47, 48]. In contrast, although SDTBCo introduces additional Co coordination sites that can adsorb O_2_, the calculated charge‐transfer values for O_2_ at these sites are positive, indicating inefficient electron donation and negligible intrinsic O_2_ activation capability. These results demonstrate that the enhanced O_2_ activation observed in SDTBCo does not originate from an increased density of metal adsorption sites.

Moreover, the interaction between photoexcited excitons and O_2_ was evaluated by comparing PL spectra recorded under N_2_ and O_2_ atmospheres. Upon O_2_ introduction, SDTBCo exhibits the most pronounced PL quenching among the three COFs (Figure 2a–c), with the anti‐Stokes emission peaks (PL2 and PL3) showing substantially stronger suppression than the intrinsic PL1 peak. This behavior indicates that a larger fraction of excitons in SDTBCo undergo dissociation or energy transfer and actively participate in O_2_ activation processes [49]. Consistently, TRPL measurements show that the excited‐state lifetime of SDTBCo (Figure 2i and Table S4) decreases most significantly when switching from N_2_ to O_2_ atmosphere, followed by SDTB (Figure 2h and Table S4) and DTB (Figure 2g and Table S4) [50]. The distinct O_2_ activation capabilities of the three COFs were further corroborated by electron paramagnetic resonance (EPR) spectroscopy. Using 2,2,6,6‐tetramethylpiperidinooxy (TEMP) as the spin trap for ^1^O_2_ (Figure S12a) and 5,5‐dimethyl‐1‐pyrroline N‐oxide (DMPO) for O_2_^•–^ (Figure S12b) [51, 52, 53], SDTBCo exhibits the strongest characteristic signals among the three samples, confirming its superior ability to generate ROS. Quantitative analyses of ^1^O_2_ (Figure S12c) and O_2_^•–^ (Figure S12d) production further corroborate this observation. These results collectively demonstrate that Co single‐atom coordination markedly enhances triplet‐state dynamics and facilitates both singlet‐ and triplet‐exciton‐mediated oxygen activation.

### 2.3 Layer‐Bridging Co Coordination Enables Energy‐Level Splitting and Enhanced SOC

To gain deeper insight into the electronic origin of the enhanced triplet‐state dynamics, the band structures of the three COFs were calculated using the Vienna Ab initio Simulation Package (VASP) [54]. Compared with DTB (Figure 3a), SDTB (Figure 3b) shows a slight downward shift of the LUMO accompanied by an increased number of electronic states. In contrast, SDTBCo (Figure 3c) exhibits a markedly increased density of electronic states with pronounced level splitting near the LUMO.

**FIGURE 3:**
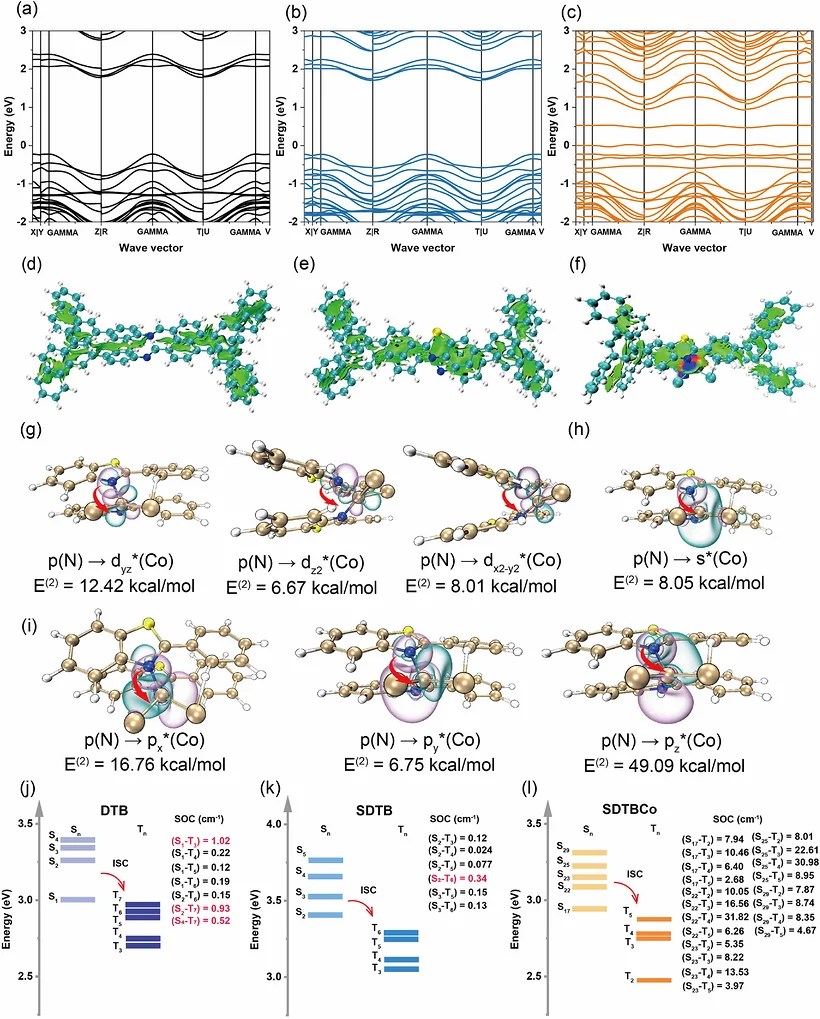
Origin of energy‐level splitting and enhanced SOC in COFs. Calculated band structures of (a) DTB, (b) SDTB, and (c) SDTBCo. IGMH analyses of (d) DTB, (e) SDTB, and (f) SDTBCo. (g–i) NBO and second‐order perturbation theory analyses of donor–acceptor (bond–antibond) interactions, together with schematic illustrations of the corresponding NBO isosurfaces (isovalue = 0.05). Energy level diagrams and SOC values of (j) DTB, (k) SDTB, and (l) SDTBCo obtained from TD‐DFT calculations.

A systematic investigation of the origin of the pronounced energy‐level splitting in SDTBCo was carried out through independent gradient model based on Hirshfeld partition (IGMH) analysis [55, 56], natural bonding orbital (NBO) analysis, and intermolecular orbital overlap calculations. IGMH analysis reveals that the interlayer interactions in DTB (Figure 3d) and SDTB (Figure 3e) are dominated by weak van der Waals forces. Sulfur incorporation within the COF backbone merely expands the van der Waals interaction region in the vicinity of the thiazole moieties, without fundamentally altering the nature of interlayer interactions. In contrast, SDTBCo (Figure 3f) exhibits pronounced Co─N coordination interactions, characterized by deep‐blue regions that are substantially stronger than hydrogen bonding interactions [57], while the remaining interlayer interactions remain comparable to those in SDTB. These results indicate that Co single‐atom coordination introduces a distinct and robust electronic coupling pathway across COF layers [58, 59].

Further insight into the specific orbitals involved in Co─N coordination was obtained through NBO analysis [60]. Upon formation of the Co─N bonds, electron donation from the N 2p orbitals to the Co antibonding d orbitals (d^*^; d*_yz_*, $d_{z^{2}}$, and $d_{x^{2}-y^{2}}$) occurs, with corresponding stabilization energies of 12.42, 6.67, and 8.01 kcal mol^−1^, respectively (Figure 3g). Additional charge transfer from N 2p orbitals to Co antibonding 4s^*^ (8.05 kcal mol^−1^; Figure 3h) and 4p^*^ orbitals (4p*_x_*, 4p*_y_*, and 4p*_z_*, with stabilization energies of 16.76, 6.75, and 49.09 kcal mol^−1^, respectively; Figure 3i) is also observed. Such extensive orbital hybridization is a prerequisite for effective orbital overlap and directly induces energy‐level splitting [61, 62, 63]. Consequently, the strong Co─N coordination‐mediated hybridization provides a fundamental electronic origin for the dense and split energy levels observed in SDTBCo.

Given that frontier orbitals directly govern charge transfer and electronic coupling, the interlayer HOMO–LUMO orbital overlap was further examined. The absolute values of the HOMO–LUMO overlap integrals between adjacent COF layers in DTB (Figure S13a), SDTB (Figure S13b), and SDTBCo (Figure S13c) are comparable, suggesting similar intrinsic π–π stacking interactions among the three systems. Notably, SDTBCo displays additional and substantially stronger orbital overlaps between the COF HOMO and Co antibonding orbitals (Figure S13d), as well as between the COF LUMO and Co bonding orbitals (Figure S13e–g), exceeding those associated with interlayer COF–COF interactions. This indicates that Co single atoms act as electronic bridges, mediating strong metal–framework orbital coupling.

Furthermore, the ISC behavior of the COFs was quantitatively analyzed through SOC calculation. Efficient ISC is essential for triplet exciton formation, which subsequently enables energy transfer to molecular O_2_ to generate ^1^O_2_ [64, 65]. In general, an effective ISC process requires both a small singlet–triplet energy gap (|Δ*E*_ST_|< 0.37 eV) and a sufficiently large SOC constant (> 0.3 cm^−1^) [66]. As shown in Figure 3j, DTB exhibits three ISC channels that satisfy these criteria, namely *S*_1_ → *T*_3_ (Δ*E*_ST_ = 0.36 eV; SOC = 1.02 cm^−1^), *S*_2_ → *T*_7_ (Δ*E*_ST_ = 0.20 eV; SOC = 0.93 cm^−1^), and *S*_4_ → *T*_7_ (Δ*E*_ST_ = 0.36 eV; SOC = 0.52 cm^−1^). In contrast, SDTB (Figure 3k) retains only one marginally effective ISC channel [*S*_1_ → *T*_3_ (Δ*E*_ST_ = 0.18 eV; SOC = 0.34 cm^−1^)], indicating that sulfur incorporation alone is insufficient to broadly activate triplet‐state pathways. Remarkably, SDTBCo (Figure 3l) displays a substantially increased number of effective ISC channels, with nearly all singlet–triplet transitions meeting the dual requirements of small Δ*E*_ST_ and large SOC. Experimentally, Δ*E*_ST_ values estimated from the energy difference between the PL and PH spectra reveal that SDTBCo (0.022 eV) possesses the smallest Δ*E*_ST_, compared with SDTB (0.11 eV) and DTB (0.095 eV). The reduced Δ*E*_ST_ facilitates an enhanced ISC rate, in good agreement with the above theoretical calculations. This pronounced enhancement can be primarily attributed to layer‐bridging Co single atoms, which introduce strong SOC and extensive energy‐level splitting [67, 68].

### 2.4 Photogenerated Carrier Separation, Transfer, and Dynamics

In situ light‐irradiated (UV) XPS analysis reveals that the binding energy of the imine nitrogen (N═C) in DTB, SDTB, and SDTBCo remains unchanged at 398.5 eV under illumination (Figure S14) relative to the dark condition (Figure S5), indicating that the imine units are not directly involved in photoinduced charge redistribution. In contrast, the binding energy of the thiazole nitrogen (N═C─S) in SDTB shifts to a lower value (400.3 eV) under illumination, accompanied by an increase in the binding energies of the sulfur species (163.9 and 165.2 eV), suggesting the occurrence of intramolecular electron transfer [69, 70]. For SDTBCo, both the thiazole nitrogen (400.5 eV) and sulfur (163.8 and 165.1 eV) exhibit reduced binding energies under illumination relative to the dark condition, whereas the binding energies of Co increase markedly (781.7, 785.9, 798.0, and 802.8 eV). Notably, upon cessation of light irradiation, the binding energies of N, S, and Co reverse their shifts observed under illumination, providing compelling evidence for reversible electron transfer driven by MLCT.

Photocurrent measurements (Figure S15a) further elucidate the role of structural modification on exciton dissociation and recombination dynamics. Relative to DTB, SDTB exhibits an enhanced instantaneous photocurrent response, indicative of improved exciton dissociation; however, the photocurrent decays rapidly, suggesting limited suppression of charge recombination [71, 72]. In contrast, SDTBCo displays both the highest photocurrent intensity and the slowest decay over an extended illumination period (up to 50 s), indicating simultaneous enhancement of exciton dissociation and long‐lived charge separation enabled by layer‐bridging Co single‐atom coordination. Moreover, electrochemical impedance spectroscopy shows that SDTBCo exhibits the smallest Nyquist semicircle (Figure S15b), suggesting the most efficient charge‐transfer kinetics from the bulk to the surface, followed by SDTB and DTB [73, 74]. The surface potential and charge distribution under light irradiation were further investigated at the nanoscale using in situ Kelvin probe force microscopy (KPFM). The changes in contact potential difference (ΔCPD) between dark and illuminated conditions for DTB, SDTB, and SDTBCo were 0.009 (Figures 4a and S16a,b), 0.249 (Figures 4b and S16c,d), and 0.430 V (Figures 4c and S16e,f), respectively. These results indicate that photoexcited electrons in SDTBCo preferentially migrate toward and accumulate on the surface under UV irradiation [75], which is highly favorable for photocatalytic reactions.

**FIGURE 4:**
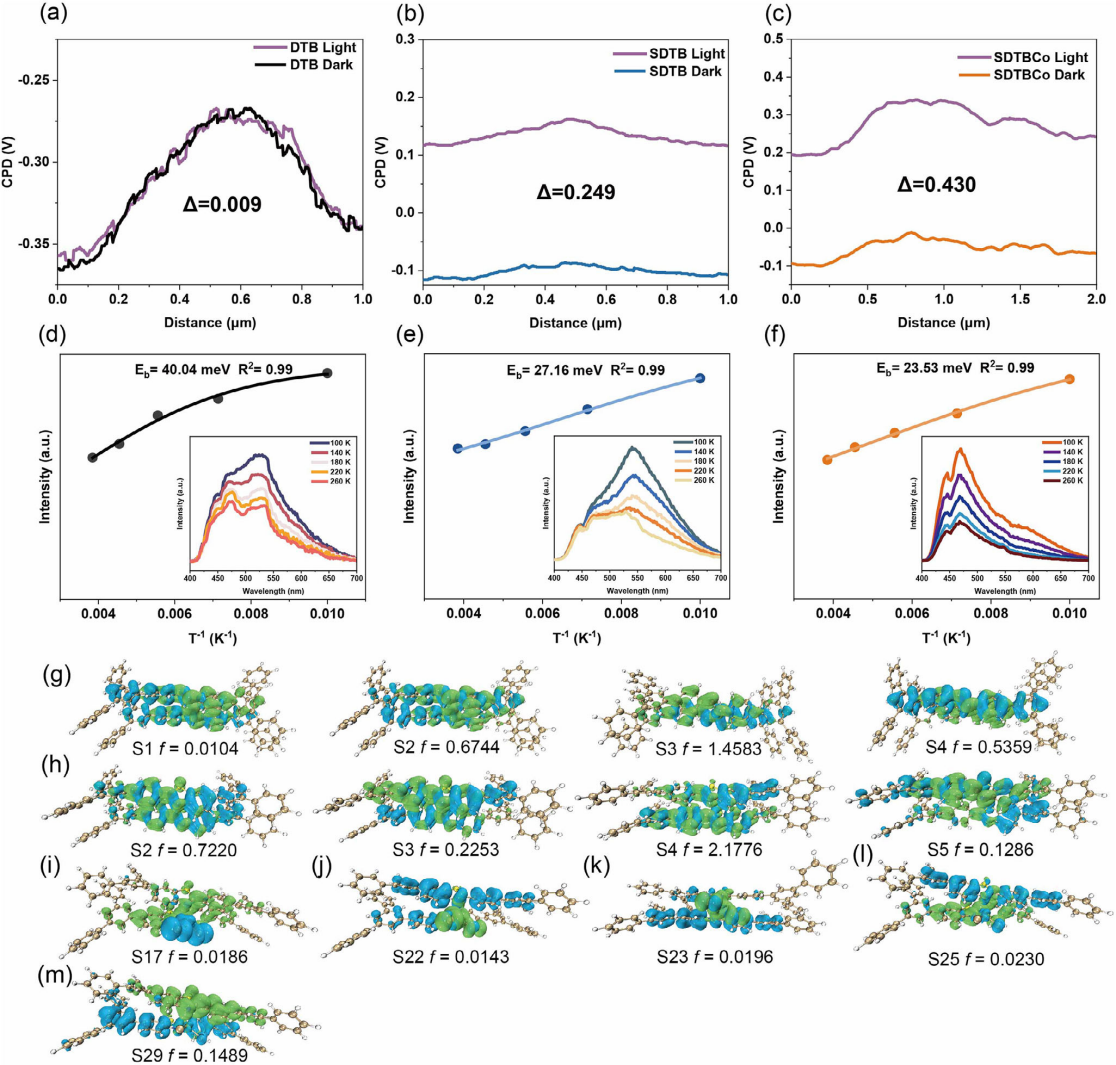
Enhanced charge‐carrier separation efficiency and its mechanistic origin. The CPD values of (a) DTB, (b) SDTB, and (c) SDTBCo measured under dark conditions and UV light irradiation. Temperature‐dependent integrated PL emission intensity in the range of 100–260 K for (d) DTB, (e) SDTB, and (f) SDTBCo (Insets: corresponding TDPL spectra). Electron‐hole distribution in the singlet excited states of (g) DTB, (h) SDTB, and (i–m) SDTBCo, where green and blue isosurfaces represent electron and hole densities, respectively (isovalue = 0.002).

Temperature‐dependent photoluminescence (TDPL) spectroscopy was employed to further probe the dynamics of photogenerated electron‐hole separation [76]. As shown in Figure 4d–f, all three COFs exhibit a monotonic decrease in PL intensity with increasing temperature. Analysis of the TDPL data yields exciton binding energies (*E*_b_) of 40.04, 27.16, and 23.53 meV for DTB, SDTB, and SDTBCo, respectively. The substantially reduced *E*_b_ of SDTBCo, falling below the thermal energy (*k*_B_ *T* ≈ 25 meV) at room temperature, signifies weakened Coulombic confinement, enabling more efficient charge separation and interfacial electron transfer.

The excited‐state charge separation was analyzed by examining the excited‐state electron‐hole distributions. Under photoexcitation, both DTB (Figure 4g) and SDTB (Figure 4h) exhibit significant spatial overlap between electron and hole densities, favoring rapid radiative or nonradiative recombination. In contrast, SDTBCo (Figure 4i–m) shows pronounced spatial separation of electrons and holes, effectively suppressing recombination and promoting population of triplet excited states rather than relaxation back to the ground state. This enhanced charge separation provides a favorable excited‐state landscape for efficient ISC.

Femtosecond transient absorption (fs‐TA) measurements were performed to explore the charge‐transfer dynamics of the COFs. Upon excitation with a 380 nm pump pulse, all COFs exhibited positive signals spread across the fs‐TA spectra (Figures 5a–c), which are assigned to excited‐state absorption (ESA) [77, 78]. ESA corresponds to the re‐excitation process of LUMO electrons to higher energy levels. Therefore, the intensity of the ESA signal reflects the population of LUMO excited states. When the intensity of the ESA signal decreases, it indicates that the number of LUMO excited states has decreased. For DTB, SDTB, and SDTBCo, the dominant ESA decay traces at the wavelengths with the highest signal intensity were selected for kinetic analysis [79, 80]. The ESA decay curves of DTB (Figure 5d) and SDTB (Figure 5e) at 535 and 525 nm, respectively, were well fitted using a double‐exponential model (Figure 5g and Table S5) [81]. This behavior indicates that the relaxation of LUMO electrons proceeds through two parallel pathways (Figure S17a): (i) trapping of high‐energy electrons into shallow energy levels (*τ*_1_) and (ii) electron transition to higher excited states (*τ*_2_). Notably, although the *τ*_2_ lifetimes of SDTB (55.8 ps) and DTB (54.3 ps) are comparable, the relative contribution of *τ*_2_ is significantly increased in SDTB. This suggests that sulfur incorporation enhances the population of high‐energy electrons in SDTB, thereby promoting O_2_ activation to generate O_2_^•–^. In contrast, the ESA signal of SDTBCo decays rapidly to zero and is accompanied by the emergence of negative signals (Figure 5f). This behavior is attributed to the strong SOC effect induced by Co single atoms, which facilitates efficient ISC from singlet to triplet states, leading to PH emission and the corresponding negative signals. Accordingly, the decay traces of SDTBCo (Figure 5f) at 530 nm (Figures 5h and S17b) were fitted using a triple‐exponential model, in which an additional component (*τ*_3_) is assigned to singlet‐to‐triplet electron transfer. Specifically, the *τ*_2_ component at 530 nm corresponds to electron transition to the higher energy levels, whereas *τ*_3_ represents ISC from singlet to triplet states. Importantly, the *τ*_2_ lifetime of SDTBCo at 530 nm (12.8 ps) is substantially shorter than those of DTB and SDTB, revealing a markedly accelerated excited‐electron transition in SDTBCo, which is favorable for the generation of O_2_^•–^. Under an O_2_ atmosphere (Figure 5i and Figure S18), the relative contribution of *τ*_2_ at 530 nm for SDTBCo is further increased (Table S5), indicating that O_2_ facilitates the capture of excited‐state electrons.

**FIGURE 5:**
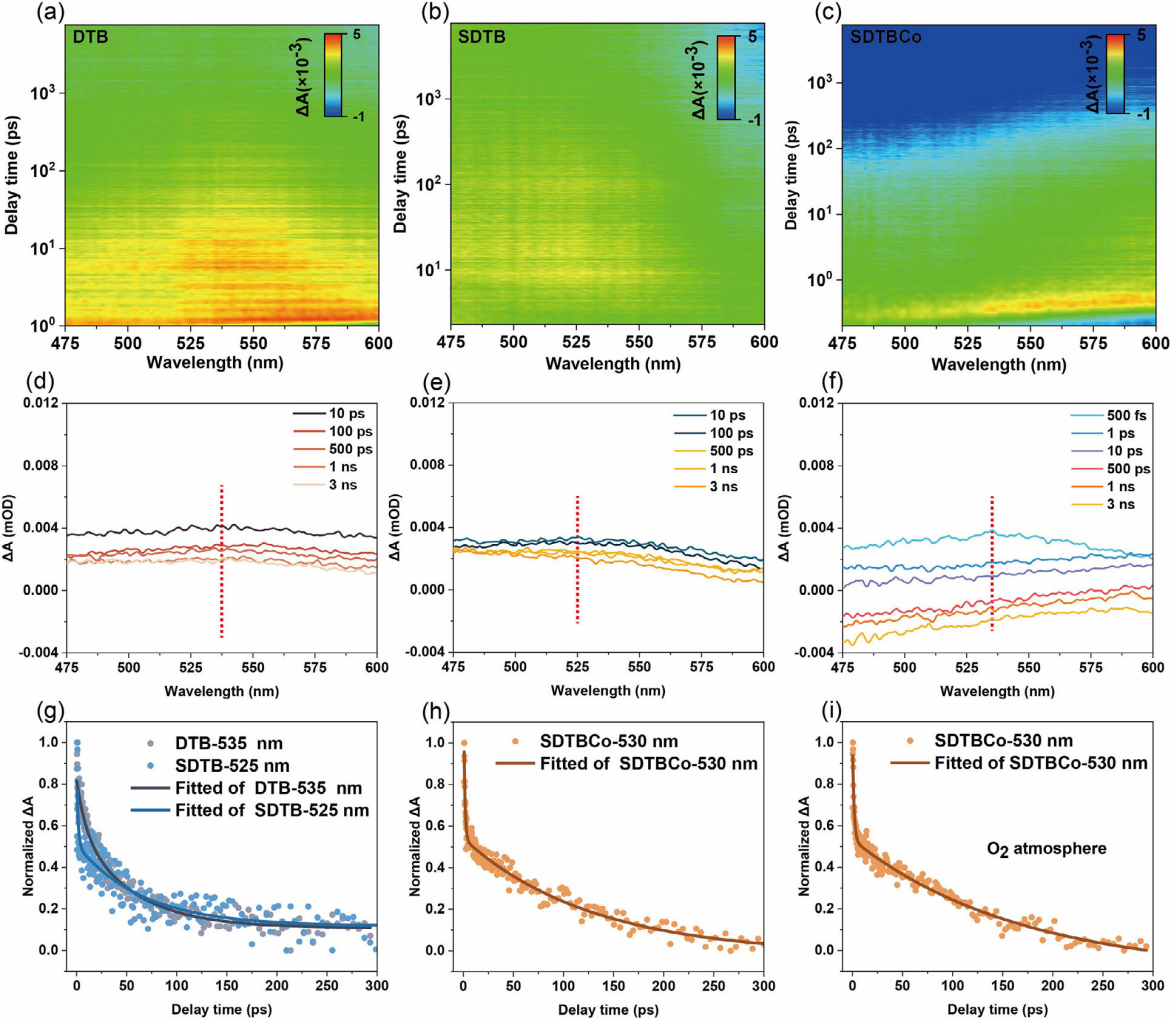
Photogenerated charge carrier dynamics probed by fs‐TA spectroscopy. Pseudocolor fs‐TA contour maps of (a) DTB, (b) SDTB, and (c) SDTBCo. Corresponding fs‐TA spectra of (d) DTB, (e) SDTB, and (f) SDTBCo at selected delay times. (g) Normalized fs‐TA decay curves of DTB at 535 nm and SDTB at 525 nm. Normalized fs‐TA decay curves of SDTBCo at 530 nm under (h) N_2_ atmosphere and (i) O_2_ atmosphere.

### 2.5 Photocatalytic H_2_O_2_ Production and Tandem C─N Coupling

The direct utilization of photocatalytic products such as H_2_O_2_ is expected to advance photocatalytic research toward practical industrial applications [82, 83, 84, 85]. Benefiting from markedly enhanced charge separation and triplet‐state activation, SDTBCo delivers superior photocatalytic performance in H_2_O_2_ production and tandem C─N coupling compared with DTB and SDTB (Figure 6a). As shown in Figure 6b, the H_2_O_2_ production rates over DTB, SDTB, and SDTBCo reach 8.5, 14.3, and 26.5 mmol g^−1^ h^−1^, respectively, while the yields for the C─N coupling between benzaldehyde (BAL) and morpholine (MOL) are 42%, 65%, and 88% (Figure 6c). Notably, SDTBCo exhibits the highest activity for both H_2_O_2_ generation and C─N coupling. The apparent quantum efficiency (AQY) values of SDTBCo are 16.3%, 13.9%, and 5.1% under monochromatic irradiation at 420, 450, and 500 nm, respectively (Figure S19). To decouple the contribution of Co coordination from sulfur incorporation, a Co‐coordinated DTB (DTBCo) was also evaluated. DTBCo achieves an H_2_O_2_ production rate of 12.3 mmol g^−1^ h^−1^ and a C─N coupling yield of 61% (Figure S20), both of which are substantially higher than those of pristine DTB, highlighting the effectiveness of single‐atom Co coordination in enhancing the photocatalytic performance of COF‐based catalysts. Furthermore, the FT‐IR spectrum, XRD pattern, and FESEM image of SDTBCo after the photocatalytic reaction remain essentially unchanged compared with those of the fresh catalyst, confirming its excellent structural stability and photocatalytic durability (Figure S21).

**FIGURE 6:**
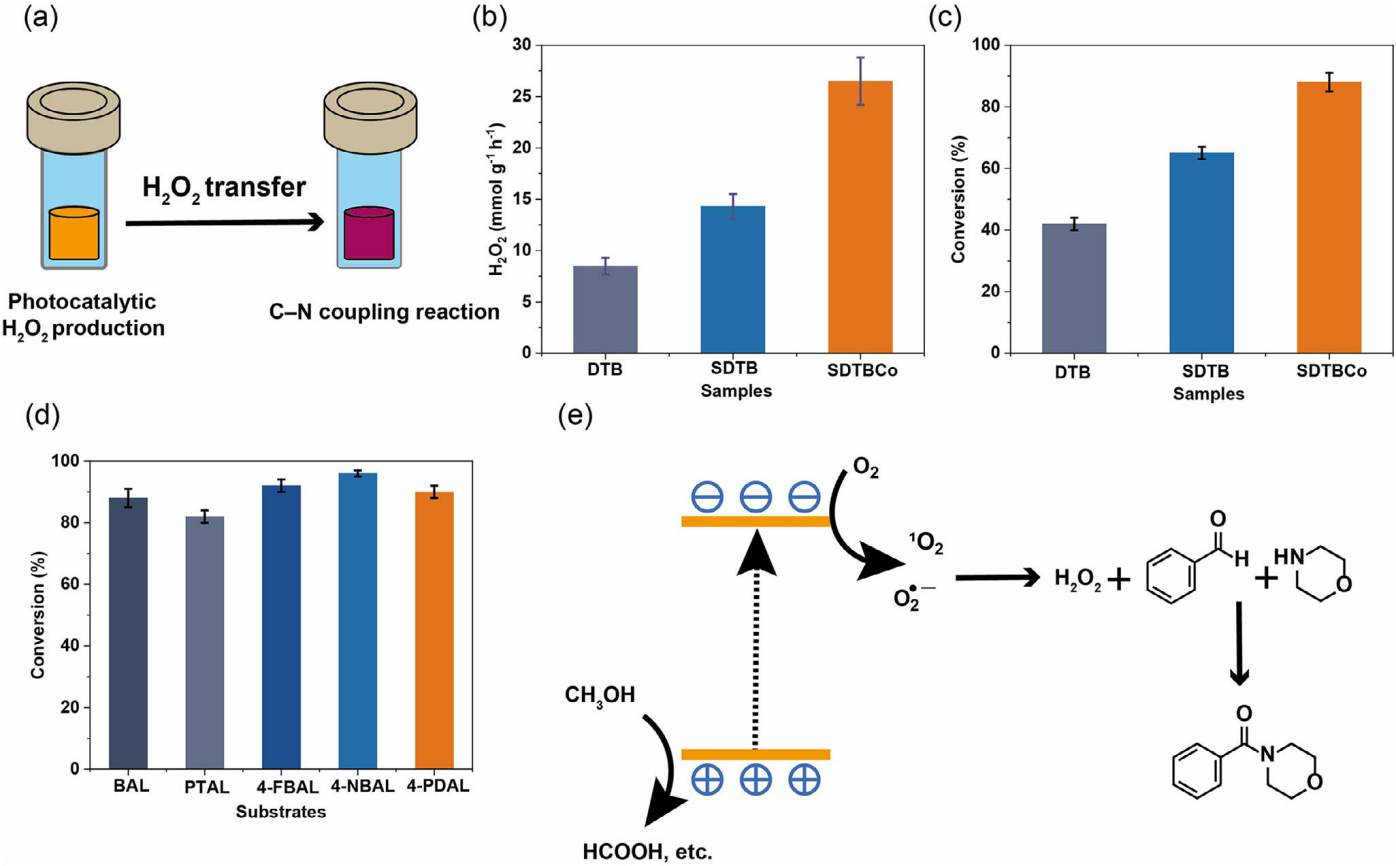
Enhanced photocatalytic performance and tandem C─N coupling reaction enabled by Co single‐atom coordination. (a) Schematic of H_2_O_2_ production and tandem C─N coupling reaction. (b) Photocatalytic H_2_O_2_ production rates and (c) C─N coupling conversions between BAL and MOL over DTB, SDTB, and SDTBCo. (d) C─N coupling conversions of MOL with various aldehyde substrates using H_2_O_2_ produced by SDTBCo. (e) Schematic illustration of the photocatalytic H_2_O_2_ production and tandem C─N coupling between BAL and MOL over the COFs.

Photocatalytic H_2_O_2_ production over these COFs proceeds via the O_2_ reduction reaction (ORR) pathway, as evidenced by controlled atmosphere experiments and scavenging tests. Replacing pure O_2_ with air or omitting methanol from the reaction system markedly decreased the H_2_O_2_ production efficiency, whereas negligible H_2_O_2_ was detected under an argon atmosphere (Figure S22a). Furthermore, isotope‐labeling analysis confirms that molecular oxygen serves as the oxygen source (Figure S22b), while methanol functions as the sacrificial agent during photocatalytic H_2_O_2_ production process. The addition of p‐benzoquinone (p‐BQ, O_2_^•–^ scavenger) or sodium azide (SA, ^1^O_2_ scavenger) severely suppressed both H_2_O_2_ production over SDTBCo (Figure S22c), indicating that these reactions are jointly mediated by O_2_^•–^ and ^1^O_2_ species, with O_2_^•–^ being further converted to ^1^O_2_ via hole oxidation. In addition, isopropanol (IPA), silver nitrate (AgNO_3_), and ethylenediaminetetraacetic acid disodium salt (EDTA‐2Na) were employed as scavengers for hydroxyl radicals (·OH), electrons (e^−^), and holes (h^+^), respectively, confirming that photogenerated electrons and protons are essential for the ORR process. The generality of this tandem strategy was further demonstrated by replacing BAL with structurally diverse aldehydes, including p‐tolualdehyde (PTAL), 4‐fluorobenzaldehyde (4‐FBAL), 4‐nitrobenzaldehyde (4‐NBAL), and 4‐pyridinecarboxaldehyde (4‐PDAL), all of which afforded satisfactory C─N coupling conversions (Figure 6d). The C─N coupling products were identified by gas chromatography–mass spectrometry (GC–MS) (Figure S23).

The intermediates involved in the ORR process were monitored by in situ diffuse reflectance infrared Fourier transform spectroscopy (DRIFTS). Upon UV irradiation, new vibrational features emerge at 939 and 1136 cm^−1^, corresponding to ^*^O─O species, while bands at 1275 and 1398 cm^−1^ are attributed to ^*^O─OH and ^*^HO─OH intermediates, respectively (Figure S24) [86, 87], indicating the formation of ROS during photocatalysis. Based on the above results, a possible photocatalytic mechanism over the COFs is proposed. Upon photoexcitation, electrons in the LUMO of the COFs reduce molecular O_2_ to generate O_2_^•–^ and ^1^O_2_, promoting the formation of H_2_O_2_. Meanwhile, holes in the HOMO of the COFs oxidize methanol, with formic acid identified as the oxidation product by nuclear magnetic resonance spectroscopy (Figure S25). The in situ generated H_2_O_2_ is subsequently transferred to drive the tandem C─N coupling reaction (Figure 6e).

## 3 Conclusion

In summary, we have developed an effective strategy to enhance the photocatalytic H_2_O_2_ production performance of COFs via layer‐bridging coordination of Co single atoms, which promotes the formation and utilization of triplet excited states. Importantly, the in situ generated H_2_O_2_ was further employed to drive a tandem C─N coupling reaction. We demonstrate that Co─N coordination between adjacent COF layers overcomes the intrinsically weak orbital interactions associated with van der Waals stacking, constructing efficient charge‐transport pathways within the crystalline framework. Both experimental characterizations and theoretical calculations confirm that the introduction of Co single atoms induces pronounced energy‐level splitting and substantially enhances SOC, thereby accelerating ISC from singlet excited states to long‐lived triplet states. As a consequence of the regulated excited‐state dynamics, the activation of molecular oxygen toward ROS, including ^1^O_2_ and O_2_^•–^, is significantly enhanced. This leads to a high photocatalytic H_2_O_2_ production rate of 26.5 mmol g^−1^ h^−1^ and an excellent tandem C─N coupling yield of 88% over SDTBCo. Distinct from conventional intralayer modifications, this layer‐bridging single‐atom engineering strategy enables efficient exploitation of interlayer electronic states in crystalline porous polymers. More broadly, this work establishes fundamental design principles for activating triplet excitons in COFs, providing new opportunities for the rational development of high‐performance photocatalysts for solar‐to‐chemical energy conversion.

## Author Contributions

**Guoen Tang**: investigation. **Jianjun Zhang**: data curation, investigation. **Guijie Liang**: data curation, investigation. **Jinfeng Zhang**: data curation, formal analysis. **Chuanbiao Bie**: conceptualization, writing – original draft. **Jiaguo Yu**: conceptualization, writing – review and editing, funding acquisition. **Hermenegildo García**: conceptualization, writing – review and editing.

## Conflicts of Interest

The authors declare no conflicts of interest.

## Supporting information




**Supporting File**: anie74270‐sup‐0001‐SuppMat.docx.

## Data Availability

The data that support the findings of this study are available from the corresponding author upon reasonable request.
