# Supplementary material for: Activating Triplet Excitons in COFs via Layer‐Bridging Co Single Atoms for Photocatalytic H2O2 Production and Tandem C─N Coupling
Source: Angew Chem Int Ed Engl. 2026 Aug 19;65(42):e1580812. doi: 10.1002/anie.1580812 (PMC13653580; doi:10.1002/anie.1580812)
Supplement: Supplementary file 1 [file ANIE-65-e1580812-s001.docx]

Supporting Information
©Wiley-VCH 2021
69451 Weinheim, Germany

**Activating triplet excitons in COFs via layer-bridging Co single atoms for photocatalytic H_2_O_2_ production and tandem C–N coupling**

Guoen Tang, Jianjun Zhang, Guijie Liang, Jinfeng Zhang*, Chuanbiao Bie*, Jiaguo Yu*, Hermenegildo García*

Table of Contents

1. Experimental procedures
2. Figures S1-S25
3. Tables S1-S5

Experimental Procedures

**Materials:**

All commercial chemicals and solvents were used as received without further purification. 1,3,5-tris(4-aminophenyl)benzene (98%), 1,3,5-tris(4-formylphenyl)benzene (98%), sulfur (99%), 1,2-dichlorobenzene (99.5%), 1-butanol (99.5%), acetic acid (36%), potassium hydrogen phthalate (C_8_H_5_KO_4_, 99.8%), potassium iodide (KI, 99.8%), dimethyl sulfoxide (99.8%), *N*,*N*-dimethylformamide (99.5%), tetrahydrofuran (99.5%), methanol (99.5%), and cobalt(II) chloride hexahydrate (CoCl_2_**^.^**6H_2_O) (≥99%) were purchased from Aladdin Scientific Co., Ltd.

**Synthesis of COFs**

DTB was synthesized via a Schiff-base condensation reaction. Briefly, 35.1 mg (0.1 mmol) of TB-NH_2_ and 39.0 mg (0.1 mmol) of TB-CHO were added to a 10 mL Pyrex tube and dispersed in a mixed solvent of 1,2-dichlorobenzene (*o*-DCB) and 1-butanol (*n*-BuOH) (3 mL, v/v = 1:1) by sonication for 5 minutes. Then, aqueous acetic acid (36 wt%, 0.3 mL) was added, followed by another 5-minute sonication to ensure homogeneous mixing. The reaction mixture was subjected to three freeze–pump–thaw cycles, sealed under vacuum, and heated in an oven at 120°C for 3 days. The obtained solid was naturally cooled to room temperature, thoroughly washed with *N*,*N*-dimethylformamide and tetrahydrofuran, and dried in a vacuum oven at 80°C overnight.

SDTB was prepared via intralayer sulfur incorporation using DTB as the precursor. Specifically, sulfur powder (28.85 mg, 0.9 mmol) and dimethyl sulfoxide (DMSO, 0.5 mL) were added to the DTB reaction system, while all other reaction conditions were kept identical to those used for DTB synthesis.

SDTBCo was synthesized through layer-bridging coordination of Co single atoms into SDTB. After completion of the SDTB synthesis, the reaction solvent was removed, and cobalt(II) chloride hexahydrate (CoCl_2_·6H_2_O, 71.34 mg, 0.3 mmol) together with methanol (CH_3_OH, 3 mL) was introduced into the reaction tube. The mixture was then sealed under vacuum and allowed to react for 1 day. The obtained solid was naturally cooled to room temperature, thoroughly washed with *N*,*N*-dimethylformamide and tetrahydrofuran, and dried in a vacuum oven at 80°C overnight.

DTBCo was synthesized following the same procedure as that used for SDTBCo, except that DTB was employed as the starting material instead of SDTB.

**Photocatalytic tests**

Photocatalytic tests were carried out in a 25-mL Pyrex flask. In each test, 5 mg of the material was added to 10 mL of an aqueous solution of 10% methanol, and a 300-W xenon lamp (*λ* > 420 nm) was used as the light source. After a 3-h reaction, 1 mL of the reaction mixture was withdrawn and filtered through a 0.45-μm filter membrane. The concentration of H_2_O_2_ was determined by adding 1 mL of 0.1 M potassium hydrogen phthalate (C_8_H_5_KO_4_) and 1 mL of 0.4 M potassium iodide (KI) aqueous solution to the sample, allowing it to stand for 1 h. The H_2_O_2_ oxidized the iodide anions (I^−^) to triiodide anions (I_3_^−^) under acidic conditions, which exhibited a distinctive absorption peak at around 350 nm. The peak intensity was measured using UV-vis spectroscopy (UVmini-1280, Shimadzu, Japan) to calculate the I_3_^−^ concentration, which was then used to calculate the H_2_O_2_ concentration, according to the reaction:

H_2_O_2_ + 3I^−^ + 2H^+^ → I_3_^−^ + 2H_2_O

**Tandem C–N coupling reactions.**

Filter out the catalyst from the solution after the photocatalytic reaction using a filter membrane, and add twice the volume of ethyl acetate to the aqueous phase. Perform three extractions to remove the remaining methanol. Take 1 mL of the treated solution and add 1 mL of acetonitrile as the solvent, then add 0.2 mmol of the amine and aldehyde raw materials, and stir at 80 °C for 1 hour. After the reaction is completed, the solution is subjected to thorough extraction with ethyl acetate and then dried with anhydrous sodium sulfate, and subjected to gas chromatography–mass spectrometry (GC–MS) analysis (Agilent 5977B GC/MSD, 8890 GC, USA) for the determination of aldehyde concentration and C–N coupling products. The main parameters for the GC-MS analysis were as follows: The initial temperature of the GC column was 40 °C and gradually raised to 200 °C. The total retention time was 20 min, and the solvent delay time was 4 min.

**Characterizations**

X-ray diffraction (XRD) was performed on a Shimadzu XRD-6100 X-ray diffractometer with Cu Kα radiation. Both conventional Fourier transform infrared (FT-IR) and in situ diffuse reflectance infrared Fourier transform spectroscopy (DRIFTS) were performed on a Nicolet iS50 spectrometer (Thermo Scientific, USA). Nitrogen adsorption–desorption measurements were conducted to determine the Brunauer-Emmett-Teller (BET) specific surface area using a nitrogen adsorption apparatus (BELSORP, MINI X). Prior to BET characterization, the samples were dispersed in deionized water followed by freeze-drying treatment. High-resolution transmission electron microscopy (HR-TEM) and energy dispersive X-ray spectroscopy (EDS) were conducted on a transmission electron microscope (Thermal Fisher Talos F200X instrument). Scanning electron microscopy (SEM) images were obtained on a scanning electron microscope (Regulus 8100). The prepared samples' UV-visible diffuse reflectance spectra (DRS) were obtained on a Shimadzu UV-2600i UV-visible spectrophotometer (Japan). The surface potential (SP) was measured using a Kelvin probe force microscopy (KPFM) (SPM-9700HT). *Ex* situ and *in situ* irradiated X-ray photoelectron spectroscopy (XPS) measurements were performed using a Thermo Scientific ESCALAB QXi XPS system (USA) with 300-W Al Kα radiation. The samples' photoluminescence (PL), phosphorescence, and phosphorescence lifetime spectra were analyzed using a fluorescence spectrophotometer (F-4700, HITACHI). The time-resolved photoluminescence (TRPL) spectra were obtained using a fluorescence lifetime spectrophotometer (FLS 1000, Edinburgh, UK). Nuclear magnetic resonance (NMR) measurements were performed on a Bruker AVANCE III 400 MHz spectrometer.

The electrochemical measurements, including photocurrent, electrochemical impedance spectra (EIS), and Mott-Schottky measurements, were performed on an electrochemical workstation (CHI660C, China) using a standard three-electrode system. A platinum foil and an Ag/AgCl electrode served as the counter and reference electrodes, respectively. The working electrode was prepared by depositing a thin film of the sample on fluorine-doped tin oxide (FTO) conductive glass. Typically, 20 mg of the powder sample was dispersed in a mixture of 300 μL of ethanol and 20 μL of Nafion, followed by sonication for 30 min. The resulting suspension was then dropped onto the surface of the FTO and dried overnight at room temperature. These electrochemical tests were performed in a 0.5 M Na_2_SO_4_ solution as the electrolyte.

The X-ray absorption fine structure (XAFS) spectra of Co K-edge were collected in transmission mode on a commercial Laboratory-Based XAFS spectrometer (Table XAFS-500A, Specreation Instruments Co., Ltd.). An X-ray tube was used to generate X-rays, and the voltage and current were set to 22 kV and 20 mA. The Si (533) spherically bent crystal analyzers with a radius of curvature of 500 mm and the R250 mm Rowland circle were used to provide monochromatized X-ray beam.

Femtosecond transient absorption (fs-TA) spectra were measured on a pump-probe system (Helios Fire, Ultrafast System) with a maximum time delay of ~8 ns, controlled by a motorized optical delay line. An 800-nm ultrashort laser pulse was generated by an ultrafast optical parametric amplifier (OPerA Solo) which was excited by a regenerative amplifier (Coherent Astrella, 800 nm, 35 fs, 7 mJ, 1 kHz), seeded with a mode-locked Ti:sapphire oscillator (Coherent Vitara-S, 800 nm, 80 MHz) and pumped with an LBO laser (Coherent Evolution-50C, 1 kHz system). The main beam was converted to a 380-nm pump pulse by the optical parametric amplifier (OPerA Solo), and a neutral-density filter was used to control the average pump power at ~73 μW. The other beam was transformed into a white-light continuum (420-800 nm) by passing through a sapphire, which served as the probe pulse.

**Radical detection capture test**

For O_2_^•^^–^ detection, 2 mg of the material was added to 1 mL of methanol, and the suspension was purged with oxygen for 5 min. Then, 10 μL of 5,5-dimethyl-1-pyrroline N-oxide (DMPO) was added, and the mixture was exposed to Xe-lamp irradiation for 5 min before the electron paramagnetic resonance (EPR) test. To detect the ^1^O_2_, 2 mg of the material was added to 1 mL of acetonitrile, and the mixture was exposed to Xe-lamp irradiation for 5 min. Then, 10 μL of 2,2,6,6-tetramethylpiperidinooxy (TEMP) was added before the EPR test. The EPR spectra were obtained on an EPR spectrometer (MEXnano, Bruker, UK) with a modulation frequency of 100 kHz and a microwave power of 25 mW.

**Radical evolution test**

The concentration of O_2_^•-^ was quantified by monitoring the reduction of Cytochrome C using UV-vis spectroscopy. After photocatalysis, 1 mL of the suspension was filtered through a 0.45-μm membrane to remove the photocatalysts. The formation of O_2_^•-^ was evaluated by the decrease in the characteristic absorption of Cytochrome C at 409 nm. The mole ratio between generated O_2_^•-^ and reacted Cytochrome C was assumed to be 1:1, according to the following reaction:

O_2_^•-^ + Cytochrome C^3+^→ Cytochrome C^2+^ + O_2_

The concentration of ^1^O_2_ was determined by monitoring the degradation of 1,3-diphenylisobenzofuran (DPBF) via UV-vis spectroscopy. After reaction, 1 mL of suspension was similarly filtered through a 0.45-μm membrane to remove the photocatalyst. The generation of ^1^O_2_ was quantified by the decrease in DPBF absorption at 410 nm. A 1:1 molar stoichiometry between ^1^O_2_ and DPBF consumption was assumed based on the following reaction:

**Isotope-labeling analysis**

The experiment was conducted in a 10 mL headspace injection bottle. In a typical photocatalytic reaction, 2 mg of the sample was dispersed in 1 mL of an aqueous H_2_^18^O solution containing 10 vol% methanol. The system was injected with high-purity oxygen through a vacuum pump, then exposed to a 300 W xenon lamp (*λ* > 420 nm) for 2 h. Upon completion of the reaction, MnO_2_ was added, and the mixture was ultrasonicated for 5 min to decompose the generated H_2_O_2_ into O_2_. The evolved gas was then analyzed using a GC–MS (Agilent 5977B GC/MSD, 8890 GC, USA).

**Computational details**

Density functional theory (DFT) calculations were performed using the Gaussian 16 software package, the ORCA quantum chemistry program package, and the Vienna Ab initio Simulation Package (VASP).

The ground-state geometry optimizations of COFs were performed at the PBE0-D3BJ/def2-SVP level. Without further relaxation, the excited state structures at the TD-PBE0-D3/def2-SVP level were calculated from the optimized ground state structures. The overlap integral of orbitals obtained through excited-state analysis was computed using the Multiwfn software.

The Generalized Gradient Approximation (GGA) described the exchange-correlation interaction with the Perdew-Burke-Ernzerhof‌ (PBE) functional. The energy cut-off was set at 450 eV. The convergence threshold for geometry optimization was set to 10^-5^ eV for energy and 0.03 eV/Å for force. A vacuum of 25 Å was used to construct surface models, thereby eliminating interactions between periodic structures.

For calculations requiring periodic boundary conditions, such as band structure calculations, we employed a periodic bulk model. In contrast, for charge transfer analysis and electronic structure calculations that do not require periodicity, a bilayer cluster model was constructed to explicitly describe the Co-mediated interlayer-bridging configuration. This model reasonably captures the local coordination environment and the essential structural features of the interlayer-bridged architecture, enabling reliable investigation of the charge transfer behavior induced by Co coordination.

Figures S1-S25

**Figure S1.** **The refined crystal structure of COFs.** (a) DTB, (b) SDTB, and (c) SDTBCo.

**Figure S2. BET analysis of COFs**. The N_2_ adsorption–desorption isotherms of (a) DTB, (b) SDTB, and (c) SDTBCo. The corresponding pore size distributions of (d) DTB, (e) SDTB, and (f) SDTBCo.

**Figure S3.** **FESEM images of COFs.** (a) DTB, (b) SDTB, and (c) SDTBCo.

**Figure S4.** **FT-IR spectra** of raw materials and COFs.

**Figure S5. XPS spectra** of (a) N 1s (DTB), (b) N 1s (SDTB), (c) S 2p (SDTB), (d) N 1s (SDTBCo), (e) S 2p (SDTBCo), (f) Co 2p (SDTBCo).

**Figure S6. HR-TEM analysis.** HR-TEM images of (a,d) DTB, (b,e) SDTB, and (c,f) SDTBCo; (g) Elemental mapping of SDTBCo.

**Figure S7. EXAFS analysis.** (a) The EXAFS R-space fitting curves of Co foil. The EXAFS k-space fitting curves of (b) Co foil and (c) SDTBCo. (d) The Co-containing structure used for the EXAFS fitting of SDTBCo (brown represents carbon atoms, yellow represents sulfur atoms, light gray represents nitrogen atoms, blue represents cobalt atoms, green represents chlorine atoms, and pinkish-white represents hydrogen atoms).

**Figure S8**. **Optical bandgap analysis of COFs.** (a) UV-vis DRS and (b) Tauc plots of COFs. Mott-Schottky plots of (c) DTB, (d) SDTB, (e) SDTBCo. (f) Band structures of COFs.

**Figure S9.** **Excitation and relaxation pathways** involving singlet excited states (S_0_→S_1_→S_n_).

**Figure S10. Phosphorescence spectra** of DTB, SDTB, and SDTBCo.

**Figure S11. O_2_ adsorption energies and charge transfer values** of (a) DTB, (b-c) SDTB, and (d-f) SDTBCo.

**Figure S12. EPR spectra** of (a) TEMP-^1^O_2_, (b) DMPO-O_2_^•–^. **Radical yields** of (c) ^1^O_2_, (d) O_2_^•–^.

**Figure S13. Orbital overlap analysis** of the interlayer HOMO-LUMO of (a) DTB, (b) SDTB, and (c) SDTBCo (isovalue = 5 × 10^–6^) with corresponding integral overlap values. (d) Orbital overlap between the COF HOMO and Co antibonding orbitals (isovalue = 5 × 10^–5^). (e-g) Orbital overlaps between the COF LUMO and Co bonding orbitals (isovalue = 5 × 10^–5^).

**Figure S14. *In situ* XPS spectra** of (a) N 1s (DTB), (b) N 1s (SDTB), (c) S 2p (SDTB), (d, g) N 1s (SDTBCo), (e, h) S 2p (SDTBCo), and (f, i) Co 2p (SDTBCo).

**Figure S15.** (a) **Photocurrent** **responses** and (b) **EIS Nyquist plots** of COFs.

**Figure S16.** **CPD maps** of (a–b) DTB, (c–d) SDTB, (e–f) SDTBCo measured under dark conditions and UV light irradiation.

**Figure S17. Decay pathways of photogenerated electrons** in (a) DTB and SDTB, and (b) SDTBCo.

**Figure S18. Pseudocolor map and** **fs-TA spectra** of SDTBCo under O_2_ atmosphere.

**Figure S19. The wavelength-dependent apparent quantum efficiency** of SDTBCo.

**Figure S20. Photocatalytic H_2_O_2_ production rates and C–N coupling** **conversions** between BAL and MOL over DTBCo.

**Figure S21. Structural characterization after the reaction.** (a) FT-IR and (b) XRD spectra of SDTBCo before and after reaction. (c) FESEM image of SDTBCo after reaction.

**Figure S22.** **H_2_O_2_ yield, isotopic analysis, and radical-capture tests.** (a) H_2_O_2_ yield over SDTBCo under control experiments. (b) Mass spectra of O_2_/Ar signal and total ion chromatography. (c) H_2_O_2_ yield over SDTBCo in the presence of different scavengers.

**Figure S23.** **Identification of C–N coupling products by GC–MS.** Mass spectra of the C–N coupling products between (a) BAL and MOL, (b) PTAL and MOL, (c) 4-FBAL and MOL, and (d) 4-NBAL, and (e) 4-PDAL and MOL.

**Figure S24.** ***In-situ* DRIFTS spectrum.** *In-situ* DRIFTS spectrum of the SDTBCo under light illumination.

**Figure S25. NMR spectrum.** The NMR spectrum of the photocatalytic reaction solution. The signal at δ = 8.11 ppm (highlighted in red) is assigned to the proton of formic acid.

Tables S1-S5

**Table S1.** *S*_BET_, pore volume (*V*_pore_), and average pore diameter (*d*_pore_) of DTB, SDTB, and SDTBCo.

| Samples | *S*_BET_ (m^2^/g) | *V*_pore_ (cm^3^/g) | *d*_pore_ (nm) |
| --- | --- | --- | --- |
| DTB | 557 | 0.41 | 2.07 |
| SDTB | 549 | 0.40 | 2.30 |
| SDTBCo | 548 | 0.39 | 2.71 |

**Table S2.** Curve-fitting parameters for the refinement of Co K-edge EXAFS of SDTBCo.

| Path | *N^a^* | R (Å)*^b^* | *σ*^2^ *(*Å^2^*)^c^* | R factor |
| --- | --- | --- | --- | --- |
| Co-N | 2 | 2.03±0.04 | 0.003±0.002 | 0.03 |
| Co-Cl | 2 | 2.31±0.01 | 0.002±0.001 |  |

*^a^N*: Coordination number. *^b^R*: Interatomic distance. *^c^σ*^2^: Debye-Waller factor (mean squared relative displacement, MSRD). *R* factor: Goodness of fit. S_0_^2^ = 0.90 was fixed at the value for the Co foil measured as a reference. Δ*E*_0_ (Inner potential correction, ‒5.29 eV) was refined as a global fit parameter for the sample. Coordination numbers were fixed at integer values to give physical meaning to the values for *σ*^2^. Data range: 2.0 < k< 9.0 Å^-1^. 1.0< R<2.5 Å. The total number of independent data points is 6.5.

**Table S3 .** Phosphorescence lifetime profiles of DTB, SDTB, and SDTBCo.

| Sample | *τ*_1_ (ms) | *A*_1_ (%) | *τ*_2_ (ms) | *A*_2_ (%) | *τ*_f_ (ms) |
| --- | --- | --- | --- | --- | --- |
| DTB | 0.43 | 77 | 4.29 | 23 | 1.32 |
| SDTB | 0.32 | 67 | 5.12 | 33 | 1.90 |
| SDTBCo | 0.46 | 67 | 7.25 | 33 | 2.70 |

Notes: *τ*_1_, *τ*_2_ represent fitted lifetime; *A*_1_, *A*_2_ represent the corresponding percentage of the fitted lifetimes; *τ*_f_ represents the overall phosphorescence lifetime.

**Table S4 .** Fluorescence lifetime profiles of DTB, SDTB, and SDTBCo.

| Sample | Atmosphere | *τ*_1_ (ns) | *A*_1_ (%) | *τ*_2_ (ns) | *A*_2_ (%) | *τ*_3_ (ns) | *A*_3_ (%) | *τ*_f_ (ns) |
| --- | --- | --- | --- | --- | --- | --- | --- | --- |
| DTB | N_2_ | 0.80 | 13 | 4.32 | 73 | 15.16 | 14 | 5.38 |
|  | O_2_ | 1.34 | 29 | 3.32 | 70 | 10.28 | 1 | 2.82 |
| SDTB | N_2_ | 0.82 | 5 | 2.68 | 42 | 13.54 | 53 | 8.34 |
|  | O_2_ | 0.38 | 25 | 2.34 | 55 | 11.76 | 20 | 3.73 |
| SDTBCo | N_2_ | 0.41 | 0.1 | 4.74 | 55.9 | 18.56 | 44 | 10.82 |
|  | O_2_ | 0.32 | 1 | 3.89 | 94 | 8.04 | 5 | 4.06 |

Notes: *τ*_1_, *τ*_2_, represent fitted lifetimes; A_1_, A_2_, A_3_ represent the corresponding percentage of the fitted lifetimes; *τ*_f_ represents the overall fluorescence lifetime.

**Table S5 .** Fitted lifetime parameters obtained from the decay curves of the samples.

| Sample | Wavelength (nm) | *τ*_1_ (ps) | *A*_1_ (%) | *τ*_2_ (ps) | *A*_2_ (%) | *τ*_3_ (ps) | *A*_3_ (%) | |
| --- | --- | --- | --- | --- | --- | --- | --- | --- |
| DTB | 535 | 2.1 | 35.9 | 54.3 | 64.1 |  |  | |
| SDTB | 525 | 1.5 | 30.0 | 55.8 | 70.0 |  |  | |
| SDTBCo-N_2_ | 530 | 1.0 | 5.4 | 12.8 | 34.4 | 150.1 | 60.2 | |
| SDTBCo-O_2_ | 530 | 1.5 | 2.3 | 15.6 | 44.4 | 125.7 | 53.3 |  |

Notes: *τ*_1_, *τ*_2_, *τ*_3_ represent fitted lifetimes; *A*_1_, *A*_2_, *A*_3_ represent the corresponding percentage of the fitted lifetimes.
